# Dynamic Model Updating for Bridge Structures Using the Kriging Model and PSO Algorithm Ensemble with Higher Vibration Modes

**DOI:** 10.3390/s18061879

**Published:** 2018-06-08

**Authors:** Shiqiang Qin, Yazhou Zhang, Yun-Lai Zhou, Juntao Kang

**Affiliations:** 1 School of Civil Engineering and Architecture, Wuhan University of Technology, Wuhan 430070, China; shiqiangqin@whut.edu.cn (S.Q.); yazhouzhang@whut.edu.cn (Y.Z.); jtkang@whut.edu.cn (J.K.); 2 Department of Civil and Environmental Engineering, National University of Singapore, 2 Engineering Drive 2, Singapore 117576, Singapore

**Keywords:** dynamic model updating, kriging model, particle swarm optimization, higher modes, bridge structure

## Abstract

This study applied the kriging model and particle swarm optimization (PSO) algorithm for the dynamic model updating of bridge structures using the higher vibration modes under large-amplitude initial conditions. After addressing the higher mode identification theory using time-domain operational modal analysis, the kriging model is then established based on Latin hypercube sampling and regression analysis. The kriging model performs as a surrogate model for a complex finite element model in order to predict analytical responses. An objective function is established to express the relative difference between analytically predicted responses and experimentally measured ones, and the initial finite element (FE) model is hereinafter updated using the PSO algorithm. The Jalón viaduct—a concrete continuous railway bridge—is applied to verify the proposed approach. The results show that the kriging model can accurately predict the responses and reduce computational time as well.

## 1. Introduction

Vibration-based structural health monitoring (SHM) for large-scale civil structures has been an ongoing research topic in both the scientific and engineering community in recent decades [1]. By placing different types of sensors on test structures and monitoring structural dynamic behavior, vibration-based SHM provides an attractive solution for health condition evaluations and operational safety management [1,2,3,4,5,6]. The acquisition of a finite element (FE) model, or so-called benchmark model that can represent the dynamic behavior of actual structures, plays a key role in vibration-based SHM. The benchmark FE model can play several roles in vibration-based SHM, such as predicting dynamic responses, detecting potential damage, and formulating vibration control strategies. However, the initial established FE model based on design documents usually could not represent a real structure due to the simplifications in FE modeling and uncertainties in the material properties. The initial FE model has to be updated based on experimental results. Model updating algorithms can be divided into two categories: direct methods and sensitive methods. Direct methods directly update the mass and stiffness matrix based on a differential equation, while sensitive methods minimize the relative differences between analytically predicted responses and experimentally measured responses by iteratively updating structural parameters that are sensitive to these responses. Since all of the elements in the mass and stiffness matrices vary individually and the positive definiteness of these matrices cannot be guaranteed, the results of direct methods usually lack physical meaning. Sensitive methods have become the more popular choice in FE model updating. A comprehensive review of the two methods and successful applications of FE model updating can be found in [7,8,9,10,11,12,13,14].

Although numerous successful applications of FE model updating have been reported, two problems still exist: (1) difficulty in extracting the higher modes; and (2) low computational efficiency. These problems are of particular concern to the dynamic model updating of bridge structures because they are more likely to sustain local damage. For the first problem, only lower modes can be experimentally identified and are considered in FE model updating; higher modes are difficult to extract using ambient vibration tests. The ambient loads (wind, microwaves, etc.) have a narrow frequency band, and cannot excite large bridge structures. The higher modes are more sensitive to local damage, and are as valuable as the lower modes, especially for SHM. Reynders and De Roeck [15] pointed out that the frequency content of ambient forces might be narrow-banded, which means that the number of modal parameters that can be determined from ambient vibration testing may be limited. Farrar et al. [16] summarized the excitation methods for a bridge structure, and found that it is unknown whether the ambient excitation source provides input at the frequency of interest. Brownjohn et al. [17] found that a key issue in FE model updating is experimentally obtaining sufficient amount modal parameters with acceptable accuracy. Moaveni and Conte [18] claimed that modal curvature is an index that is very sensitive to local damage, while the higher modes have higher curvatures. In summary, it is important to identify higher modes and consider them in FE model updating. In terms of computational efficiency, the relationship between responses and structural parameters is implicit; the analytical responses in each iteration step can only be predicted using a complex FE model, thus leading to high computational cost. The use of a surrogate model that explicitly describes the relationship between responses and structural parameters can reduce the computational time. Marwala [19] first introduced this idea into FE model updating, where a multi-layer perceptron was employed to approximate the implicit relationship, and the FE model of an H-type structure was updated with good accuracy. Afterwards, many researchers have investigated surrogate models in FE model updating. Deng and Cai [20] updated a concrete bridge based on the response surface method and a genetic algorithm. In their study, the second polynomial was used as the surrogate model. Shan and Li [21] updated a cable-suspension bridge prototype based on a surrogate model and the substructure method. Ren and Chen [22] systematically explored the response surface method in FE model updating, and by simply using a steel I-beam and a full-scale concrete bridge, they found that the response surface model can serve as a surrogate model for FE model updating. In addition to the response surface method, the kriging model is also used as a surrogate model. The kriging model can produce superior modeling of the random errors in data samples and the highly nonlinear relationship between responses and structural parameters [23]. The kriging model has been applied in many research areas such as geology, optimization, and aerospace engineering [24]. However, there are only a few instances in the literature where the kriging model is used in FE model updating, and the most relevant studies mainly focus on simple structures in the laboratory [25,26]. Further investigation is still necessary in order to determine the robustness and effectiveness of the kriging model for application to complex full-scale bridge structures.

To resolve the previously mentioned problems involved in the model updating of bridge structures, this study combines the kriging model and particle swarm optimization (PSO) using the higher vibration modes from ambient vibration tests. The outline of this study is as follows: Section 2 presents the theoretical basis for higher mode identification based on time-domain operational modal analysis (OMA) considering large-amplitude initial conditions. Section 3 explains how to establish the kriging surrogate model. Section 4 presents a basic explanation of the PSO algorithm. Section 5 summarizes the FE model updating procedure. Section 6 presents the verification of the proposed method on a full-scale concrete continuous bridge. Finally, the concluding remarks are presented.

## 2. Higher Mode Identification

The higher modes, which are more sensitive to local damage, are of high significance to be identified in experiments and used to update the dynamic FE model. However, the narrow frequency band of ambient loads cannot fully excite large-scale bridge structures. One possibility is increasing the excitation frequency content, such as through using special excitation device. However, such a special excitation device usually requests large domains and a high cost. During dynamic tests, the bridge operation is interrupted, and additional damages might be introduced to the bridge structure, as reported in [27]. Qin et al. [28] investigated whether OMA can start from large-amplitude initial conditions, as opposed to more common zero-amplitude or low-amplitude initial conditions, and can serve as an alternative method for enlarging the frequency content of the dynamic response. Large-amplitude initial conditions can appear immediately after a train passes over a railway bridge (free vibration after train passage), or by the sudden release of a heavy mass. Their study found that by considering large-amplitude initial conditions, several higher modes of a railway bridge could be extracted. A similar comparison between OMA using pure ambient vibration measurements and free vibration measurements is reported in [29,30].

The basic idea behind considering large-amplitude initial conditions in time-domain system identification methods, which is based on output correlation functions, is summarized as follows. The stochastic state space model [31] can be expressed as:

(1) $$\left\{ \begin{array}{l} x_{k+1}=Ax_{k}+w_{k}\\ y_{k}=Cx_{k}+v_{k}, \end{array}\right.$$

where $x_{k}$ and $y_{k}$ are the state vector and output measurements, and ***A*** and ***C*** are the state matrix and output matrix, respectively. Meanwhile, $w_{k}$ is the process noise and $v_{k}$ is the measurement noise, and both can be assumed to be zero-mean white noise. Substituting $x_{k}$ in the second equation of Equation (1) with the first equation of Equation (1), the output measurements, $y_{k}$, can be recursively expressed as:

(2) $$y_{k}=CA^{k}x_{0}+\sum_{m=1}^{k}CA^{k-m}w_{m-1}+v_{k},$$

where $x_{0}$ represents the initial condition. Equation (2) shows that the output measurements are composed of two parts. The first part, $y_{k}^{ini}$, is determined by the initial condition, whereas the second part, $y_{k}^{amb}$, contains the influence of ambient excitation:

(3) $$y_{k}^{ini}=CA^{k}x_{0},$$

(4) $$y_{k}^{amb}=\sum_{m=1}^{k}CA^{k-m}w_{m-1}+v_{k},$$

where the superscript “*ini”* indicates initial, and “*amb”* represents ambient. Thus, the output covariance matrix, $\Lambda_{i}$, can be derived from:

(5) $$\Lambda_{i}=\frac{1}{N}\sum_{k=0}^{N-i}y_{k+i}y_{k}^{T}=\frac{1}{N}\sum_{k=0}^{N-i}\left(y_{k+i}^{ini}+y_{k+i}^{amb}\right){\left(y_{k}^{ini}+y_{k}^{amb}\right)}^{T},$$

where *N* means the data samples. Equation (5) illustrates that when considering large-amplitude initial conditions, the output covariance is also composed of two parts. Since the output matrices (***A***, ***C***) are directly estimated by output covariance, the modal parameters extracted from the output covariance matrices are affected by large-amplitude initial conditions, which can enlarge the frequency band of excitations. Detailed theory formulations and application can refer to [28].

## 3. Kriging Model

The kriging model [32] is a half-parameterized interpolation model including one parametric and one non-parametric part that has been applied in model updating [33]; for, details one can refer to [33]. Since the relations from structural response to parameters are implicit, one needs to obtain data samples to describe their implicit relationship via the design of experiments (DOE). The kriging model is then developed through a regression analysis of the data samples. Sampling algorithms for DOE can be divided into two categories: standard designs and computer-generated designs (CGDs) [34]. Further discussion for certain categories can also refer to [33]. The standard designs, such as full factorial design (FFD) and central composite design (CCD), highlight the precision and randomness of design points, while CGDs such as Latin hypercube sampling (LHS) and uniform design (UD) mainly focus on space filling and the uniformity of design points. This study applies LHS for the DOE due to its simplicity and because it can provide randomly generated and uniformly distributed data samples in the design space. LHS, which was initially raised by McKay [35], has widely served as a multi-dimensional stratified sampling method. Every subinterval will be independently and randomly sampled only one time in LHS. 

## 4. Particle Swarm Optimization

PSO, one of the evolutionary optimization algorithms, has already been applied in numerous optimization problems [36]. It was modeled after the swarm behavior of birds, fish, and bees when searching for food. The particles fly around in a multi-dimensional search space in pursuit of an optimal location (e.g., the most fertile food location) that minimizes the specific objective function. Compared with other evolutionary algorithms, PSO has the advantages of conceptual simplicity, fewer parameters, easy programmability, and relatively low computational costs.

PSO only has two equations, which are expressed as:

(6) $$\{\begin{array}{c} x_{i}\left(t+1\right)=x_{i}\left(t\right)+v_{i}\left(t\right)\Delta t\\ v_{i}\left(t+1\right)=\omega v_{i}\left(t\right)+c_{1}\varphi_{1}\left(pb_{i}\left(t\right)-x_{i}\left(t\right)\right)+c_{2}\varphi_{2}\left(pg_{i}\left(t\right)-x_{i}\left(t\right)\right). \end{array}$$

Each particle, *i,* has a position vector, $x_{i}\left(t\right)$, and a velocity vector, $v_{i}\left(t\right)$, at generation, *t*, in the multi-dimensional space. The purpose of the first equation in Equation (x) is to update the position of particle *i* at the next generation (*t* + 1). The purpose of the second equation is to update the velocity of particle *i*. ω is inertial weight, $pb_{i}\left(t\right)$ is the current, local, optimal position discovered by particle *i*, and $pg_{i}\left(t\right)$ is the best global position found by the swarm. $c_{1}$ and $c_{2}$ are positive constants referred to as cognition and social coefficients, respectively, $\varphi_{1}$ and $\varphi_{2}$ are the random numbers uniformly distributed between [0, 1]. Using the positions of individual particles, the algorithm calculates the fitness of each particle according to the objective function, and then adjusts the position to the optimal location.

## 5. Model Updating with Kriging Model

The FE model updating is an optimization problem by adjusting the updating parameters in their design space. FE model updating minimizes the objective function that reflects the difference between the analytically predicted response and the experimentally measured response. The objective function, J(x), can be illustrated as:

(7) $$J\left(x\right)=\sum_{i=1}^{n}\alpha_{i}r_{i}\left(x\right),$$

where $x=\left(\begin{array}{cccc} x_{1} & x_{2} & \cdots & x_{k} \end{array}\right)$ indicates the updating parameters, $r_{i}\left(x\right)$ means the residual function that expresses the relative difference between the analytically predicted response and the experimentally measured response, and *n* represents the number of responses used in the FE model updating process. The frequency and the modal assurance criterion (MAC) are the most common responses used in dynamic model updating. $\alpha_{i}$ depicts the weighting factor of each response. To ensure that the updated result is physically meaningful, each updating parameter, $x_{i}$, will be set to a lower and an upper boundary ($x_{i}^{l}$, $x_{i}^{u}$). Therefore, FE model updating is a constrained optimization problem that can be described as:

(8) $$\begin{array}{ll} \min: & J\left(x\right)\\ s.t.:. & x_{i}^{l}\leq x_{i}\leq x_{i}^{u}. \end{array}$$

For FE model updating with the kriging model, the analytically predicted response in Equation (17) is derived from the kriging surrogate model rather than the complex FE model. The entire procedure for dynamic model updating can be summarized as the following steps.

Step 1: Establish the initial FE model according to the design documents;

Step 2: Perform ambient vibration tests and modal identification;

Step 3: Select the updating parameters based on a sensitivity analysis. Set a reasonable lower and upper boundary for each updating parameter;

Step 4: Establish a kriging surrogate model between the updating parameters and the response. Evaluate the accuracy of the kriging model using the root mean squared error (RMSE) value;

Step 5: Build the objective function based on Equation (17). The analytical responses are predicted by the kriging surrogate model. The experimental responses are obtained from the ambient vibration test;

Step 6: Minimize the objective function using PSO by iteratively searching through the updating parameter values in the design space;

Step 7: Analyze and evaluate the updating results.

**Figure 1:**
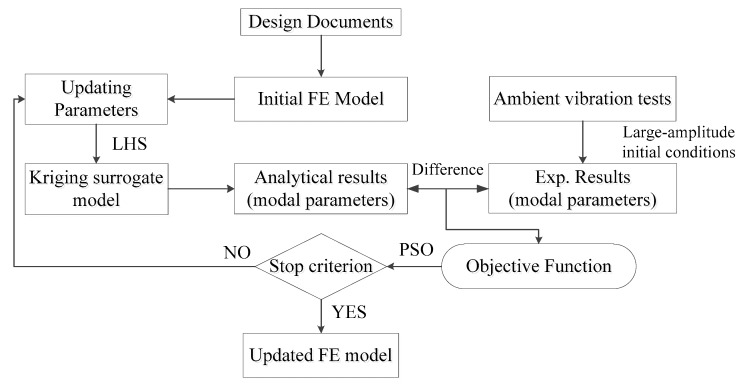
Flow chart of the finite element (FE) model updating with kriging model.

Figure 1 presents a flowchart summarizing this process.

## 6. Model Updating Verification for Jalón Viaduct

### 6.1. Bridge Description and Summary of OMA 

**Figure 2:**
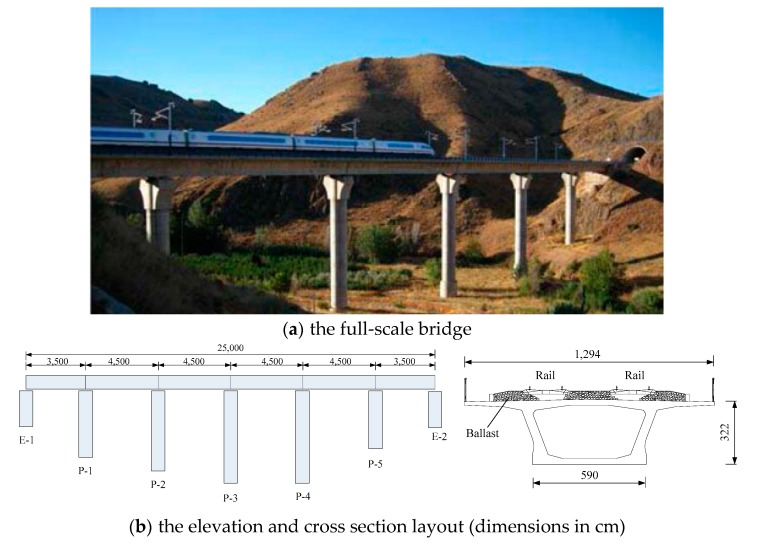
The Jalón viaduct: (**a**) the full-scale bridge; (**b**) the elevation and cross-section layout (dimensions in cm).

The Jalón viaduct (Figure 2) is a six-span prestressed concrete box girder bridge that has been applied for high-speed railways in Calatayud, Spain. The total length of the bridge is 250 m, and the layout of the span is (35 + 45 × 4 + 35) m. The bridge deck has 12.94 m in width, supporting two parallel railway lines that run in opposite directions. An initial finite element model of the Jalón viaduct is established based on ANSYS (ANSYS, Inc 2855 Telegraph Avenue, Suite 501 Berkeley, CA 94705). The box girders were modeled by Solid45 elements, and the piers were modeled by Beam4 elements. In total, 17,690 elements and 23,970 nodes were used in the initial model. The material properties were determined from design drawings. The theoretical modal analysis was performed based on the initial finite element model. 

A detailed ambient vibration test campaign using 12 wireless acceleration sensors (GeoSig GMS-18, GeoSIG Ltd, Schlieren, Switzerland) was performed to obtain the dynamic properties of the Jalón viaduct. The wireless sensors are interconnected through a time-synchronous WIFI network [37,38]. In total, accelerations at 303 points that divided into 38 setups were measured. The sampling frequency was 200 Hz and for each setup, and the sampling time was approximately 15 min. OMA was implemented hereinafter starting from both low-amplitude initial conditions (pure ambient vibration) and large-amplitude initial conditions (combined free-ambient vibration). Detailed information for the ambient vibration test and the OMA for the Jalón viaduct can refer to [28]. It was found that some higher modes can be extracted by considering the large-amplitude initial conditions induced by high-speed trains. Table 1 illustrates the lower modes that can be identified from both ambient vibration and free vibration. Table 2 shows the higher modes that can only be identified from combined free–ambient vibration. The modal phase collinearity (MPC) value is an index that reflects the smoothness of the identified mode shapes. An MPC value that is close to one is desirable, because the identified mode shape is smooth. For the identified lower modes, the experimental frequency values are close to the analytical ones, and the MPC and MAC values are all close to one; this suggests that the lower modes are very well determined. For the higher modes, the errors between the experimental and analytical values are slightly higher than that of the lower modes, and the MAC values vary from 0.499 to 0.958, meaning that the initial model has to be updated. Applying the FE model updating process to the Jalón viaduct preserves the accuracy of the lower modes, but concurrently improves the accuracy of the higher modes.

### 6.2. Updating Parameters Selection

**Figure 3:**
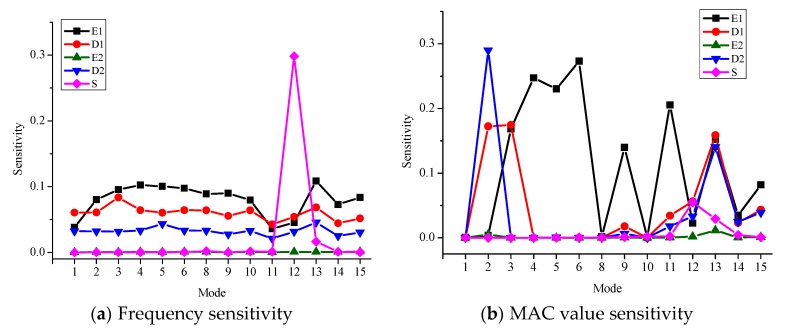
The frequency sensitivity and MAC value sensitivity of the structural parameters.

**Figure 4:**
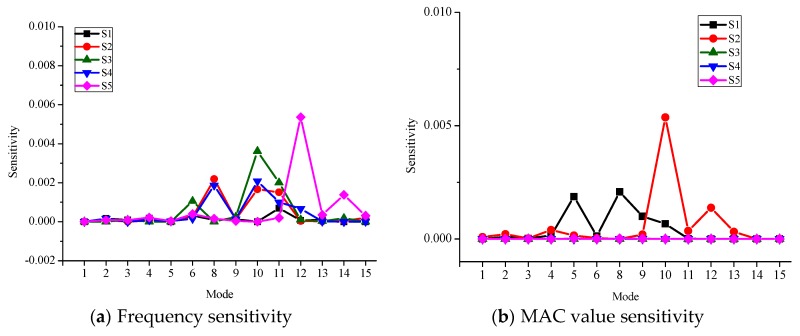
The frequency sensitivity and MAC value sensitivity of the stiffness of five supports.

Several structural parameters, including Young’s modulus of the main girder and ballast (denoted by E1 and E2), the density of the main girder and ballast (denoted by D1 and D2), and the stiffness of five supports (denoted by S) are first selected by a sensitivity analysis [22]. Figure 3 shows the frequency and MAC value sensitivity of each structural parameter. For the frequency sensitivity, both lower and higher frequencies are sensitive to E1, D1, and D2, but are insensitive to E2 and S. For the MAC sensitivity, the higher modes are more sensitive to E1, D1, and D2 compared with the lower modes. Only some of the higher modes are sensitive to E2 and S. To further examine the influence of different supports, the frequency sensitivity and MAC sensitivity to the stiffness of each support (denoted by S1-S5) were derived, and are shown in Figure 4. The results suggest that the frequencies and MAC values are most sensitive to S1 and S5, and the absolute values of both frequency and MAC sensitivities are very small. With the sensitivity analysis shown in Figure 3 and Figure 4, six structural parameters are selected as updating parameters: E1, D1, E2, D2, S1, and S5. The Young’s modulus of ballast, E2, is taken as an updating parameter, since the higher modes of the viaduct are more sensitive to it. In accordance with the design documents, the initial values of the six updating parameters are set as 4 × 10^4^ MPa, 2500 kg/m^3^, 280 MPa, 1700 kg/m^3^, 2 × 10^5^ kN/m and 2 × 10^5^ kN/m, respectively.

### 6.3. Kriging Model Establishment

The LHS-based experimental design is implemented first to establish the kriging surrogate model for the FE model of the Jalón viaduct. For each updating parameter, 800 data samples are generated by LHS. Considering the physical meaning of each parameter, the upper and lower boundaries of Young’s modulus and density are set to 1.2 and 0.8 times their initial values. The corresponding coefficients for the stiffness of support are set to 2.0 and 0.8.

**Figure 5:**
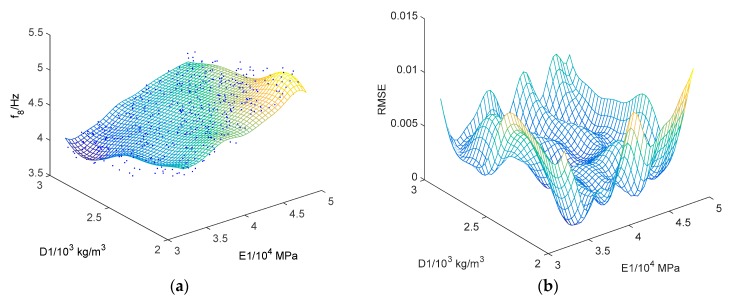
The kriging surrogate model: (**a**) *f_a,ini_ of mode 8* (4.421 Hz) in the function of D1 and E1; (**b**) the corresponding RMSE.

The kriging surrogate model, indicating the relationship between the updating parameters and modal frequencies/MAC values, is established based on a regression analysis of the data samples obtained from LHS. The Gaussian process modeled the local deviation part in Equation (7), while the global trend is simulated by a constant. In total, 14 frequencies and 14 mode shapes in the function of six updating parameters are established by using the kriging approach. Figure 5 demonstrates the typical result of *f*_a,ini_ of mode 8 (4.421 Hz) in the function of D1 and E1, along with the corresponding RMSE. The dots in Figure 5 are the data samples generated by LHS, while the kriging surrogate model generates the surface. The RMSE values are smaller than 0.01, indicating that the surrogate model is quite accurate.

### 6.4. Model Updating

In order to update the initial model of the Jalón viaduct, the objective function that quantifies the errors between the analytically predicted responses (i.e., frequencies and MAC values) and the experimentally measured ones can be expressed by:

(9) $$\mathrm{Obj}={\sum_{i=1}^{14}\left(\frac{f_{a,i}-f_{e,i}}{f_{e,i}}\right)}^{2}+{\sum_{i=1}^{14}\left(\frac{1-{\mathrm{MAC}}_{i}}{{\mathrm{MAC}}_{i}}\right)}^{2},$$

where the first part is the relative difference between analytical and experimental frequencies, while the second part is the difference between mode shapes in terms of the MAC values. The subscripts “*a*” and “*e*” represent the words “analytical” and “experimental”, respectively. “*i”* is the modal order. The analytical values are predicted by the kriging surrogate model instead of the complex FE model in order to reduce computation time. The experimental values are shown in Table 1 and Table 2. Since the higher modes are considered in model updating, all of the experimental values refer to the results extracted from the combined free–ambient vibration.

**Figure 6:**
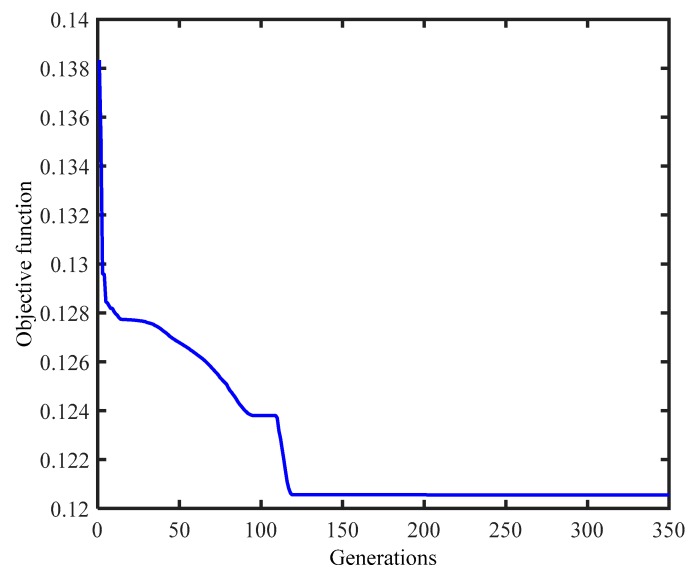
Convergence of the objective function.

Then, the PSO is applied iteratively to find the minima of the objective function in the design space. A population of 100 particles is used. The PSO parameters in Equation (16) are set to γ=0.729, $c_{1}=0.5$, and $c_{2}=1.5$ (known as default PSO variant, see [39]). The stopping criteria are set as follows: the maximum number of iterations is 2000, or the difference of the objective function value between two adjacent iterations is smaller than 10^−6^. Figure 6 illustrates the convergence of the objective function. After about 150 iterations, the objective function value decreases from 0.14 to 0.12. Since the experimental values of the lower modes agree well with the analytical values, the objective function value is relatively small. However, the higher modes are greatly enhanced after model updating, especially the modes’ shapes. This improvement will be discussed in detail in the following section.

Table 3 gives the updating parameters’ values before and after updating. The updated parameters should be in a reasonable range and physically meaningful; otherwise, it is challenging to continue updating the results. For the main girder, the Young’s modulus, E1, undergoes a slight decrease of 3.8% after updating. The density, D1, decreases by 5.6%. These changes suggest that the stiffness of the main girder in the initial FE model was overestimated. The may be because the human holes at the support and center diaphragms are ignored in the initial FE model. The prestress ducts are also neglected. For the ballast, the Young’s modulus, E2, decreased by 13.6% after updating, and the density D2 decreased by 9.4%. This decrease is because the stiffness of ballast always changes under operation. For instance, the ballast will become dense (increased stiffness) with the action of vertical load, while it can also become loose (decreased stiffness) due to regular management. The updated results may only demonstrate the real state of ballast during ambient vibration tests. For the bearing’s stiffness, S1 and S5 have increased by 64.5% and 71.5%, respectively. This may be because all of the bearings adopt same stiffness value without considering the difference in bearing type in the initial FE model. Although the change in bearing’s stiffness is plausible, the updating results still located in a reasonable range, as also reported by [20,36].

Table 4 illustrates a comparison between the modal frequencies and MAC values of the Jalón viaduct obtained from the experiment, the initial FE model, and the updated FE model as well. The differences between the analytically predicted values and the experimentally measured values are also listed.

Since the lower modes of the initial FE model already agree well with the experimental values, the accuracy of the modal frequencies remains very high after updating. The accuracy of some frequencies even increased; for instance, the relative difference decreased from 7.25% to 2.18% for the first mode and decreased from −2.92% to −1.85% for the fifth mode. Some modes’ errors slightly increased after model updating, such as mode 4 and mode 6. However, the relative errors of the frequencies of mode 4 and mode 6 after model updating are still smaller than 3%. This indicates that although model updating can improve the accuracy of the FE model, some trade-offs are inevitable [35]. On the other hand, the MAC values of the lower modes are closer to one after updating, and all of the MAC values of the lower modes are greater than 0.96, indicating that the mode shapes of the updated model are closer to the experimental values.

For the higher modes, the accuracy modal frequencies are greatly promoted. For instance, the relative difference in the modal frequency of mode 11 decreased from –7.69% to –1.18% after updating. The accuracy of mode 13 and mode 15 also improved. In addition, the MAC values of the higher modes also increased. For instance, the MAC value of mode 11 increased from 0.865 to 0.929.

**Figure 7:**
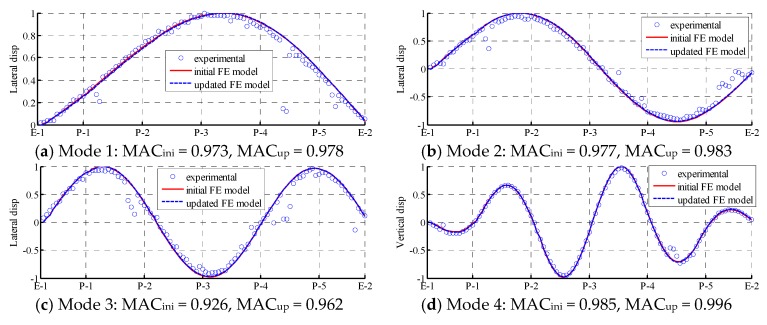

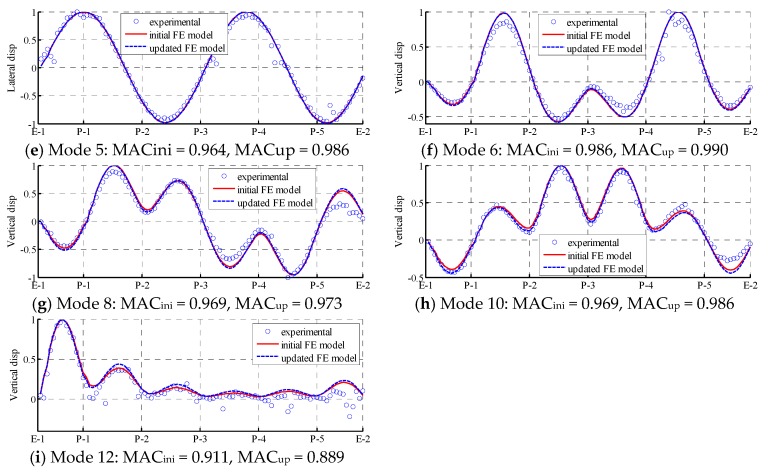
Lower modes that can be identified from pure ambient and combined free–ambient vibration: comparison of mode shapes among experimental values (dot), initial FE model (solid line), and updated FE model (dash line).

**Figure 8:**
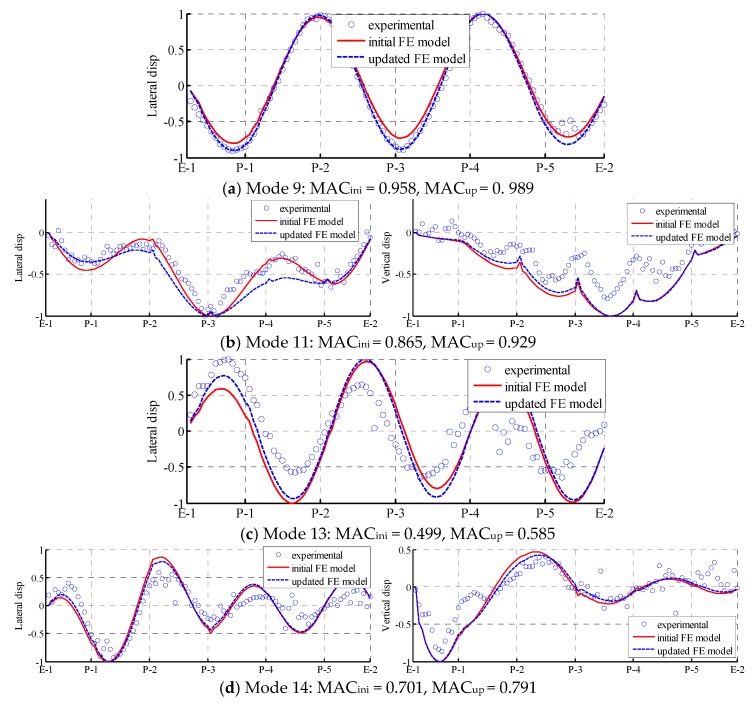

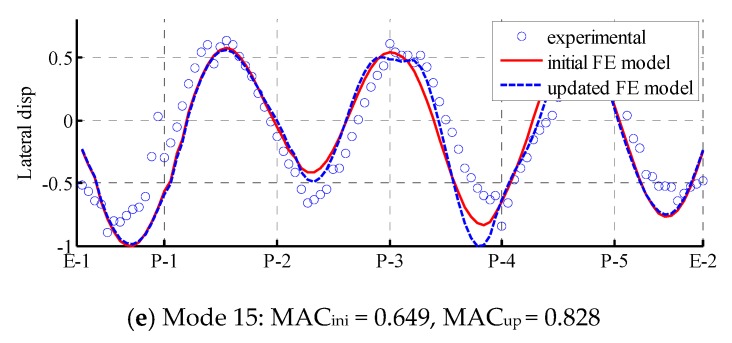
Higher modes that can only be identified from combined free–ambient vibration: comparison of mode shapes among experimental values (dot), initial FE model (solid line), and updated FE model (dash line).

Figure 7 presents a comparison of the mode shapes of lower modes from the experimental values, the initial FE model, and the updated FE model. As shown in the Figure 7, both the initial FE model and updated FE model agree well with the experimental values. Only in some local areas, such as the crests and troughs of the mode shapes, does the updated FE model not match well with the experimental test results. Figure 8 presents a similar comparison with higher modes. Even the MAC values and MPC values of higher modes are smaller than those of lower modes; the improvement in the accuracy of the mode shapes in the updated FE model still exists. For instance, in mode 9, the mode shape from the updated FE model almost completely coincides with the experimental values. Other mode shapes also demonstrate distinct degrees of improvement. The updating of these higher modes is essential in further SHM and the local damage detection of the Jalón viaduct.

In summary, the accuracy of both modal frequencies and mode shapes are improved in the updated FE model of the Jalón viaduct. The higher modes are considered in model updating to allow the FE model to better represent the dynamic behavior of the physical structure. Since the updated model agrees well with the experimental results, the updated model can serve as the baseline FE model of the Jalón viaduct.

## 7. Conclusions

This study presented a dynamic model-updating process for bridge structures based on the kriging model and PSO algorithm with higher vibration modes under large-amplitude initial conditions. The kriging model that was established based on LHS and a regression analysis of the data samples can serve as a surrogate model for a complex FE model in analytical response prediction, and the effectiveness of the proposed method was investigated in a full-scale application of a six-span railway viaduct. The following conclusions can be drawn.

The accuracy of the modal frequencies and mode shapes of higher modes are greatly improved after model updating. Therefore, the updated FE model can be used for the benchmark model of the Jalón viaduct.

The updated FE model of the Jalón viaduct agrees well with the experimental results in both lower and higher modes, which suggests that the proposed approach has good performance in bridge structural dynamic analysis. 

In terms of computational cost reduction, the proposed approach can reduce the computational cost associated with FE model updating, which implies potential further application in engineering application. 

## Figures and Tables

**Table 1:** The lower modes that can be extracted from both ambient and combined free-ambient vibration.

Mode	Type	Initial FE Model	Ambient Vibration		Free-Ambient Vibration			
		*f_a,ini_* (Hz)	*f_ea_* (Hz)	$\xi_{ea}$ (%)	*f_ef_*	$\xi_{ef}$ (%)	MPC	MAC
1	1st↔	0.601	0.648	0.75	0.648	0.69	0.94	0.973
2	2nd↔	1.182	1.238	0.81	1.219	1.34	0.96	0.977
3	3rd↔	2.201	2.230	1.00	2.186	1.53	0.85	0.926
4	1st↕	3.264	3.279	0.42	3.267	0.40	0.96	0.985
5	4th↔	3.595	3.536	1.37	3.493	0.70	0.93	0.964
6	2nd↕	3.788	3.758	0.54	3.783	0.61	0.98	0.986
8	4th↕	4.421	4.471	0.66	4.452	0.48	0.98	0.969
10	5th↕	5.015	5.056	0.57	5.045	0.62	0.97	0.969
12	6th↕	5.996	6.036	0.69	6.027	0.70	0.91	0.911

Note: ↔ stands for lateral bending modes; ↕ stands for vertical bending modes. MPC: modal phase collinearity. MAC: modal assurance criterion.

**Table 2:** The higher modes that can only be extracted from combined free-ambient vibration.

Mode	Type	Initial FE Model	Free-Ambient Vibration			
		*f_a,ini_* (Hz)	*f_ef_*	$\xi_{ef}$ (%)	MPC	MAC
9	5th↔	5.211	4.917	0.74	0.94	0.958
11	1st↺	5.717	5.309	0.73	0.85	0.865
13	6th↔	7.554	7.455	0.70	0.87	0.499
14	2nd↺	9.290	8.420	0.96	0.83	0.701
15	7th↔	10.180	9.329	0.96	0.79	0.649

Note: ↔ stands for lateral bending modes; ↺ stands for torsion modes.

**Table 3:** Comparison between initial values and updated values of updating parameters.

Updating Parameters	Initial Value	Updated Value	Difference (%)
E1 (10^4^ MPa)	4.00	3.85	−3.8
D1 (10^3^ kg/m^3^)	2.50	2.36	−5.6
E2 (10^2^ MPa)	2.80	2.42	−13.6
D2 (10^3^ kg/m^3^)	1.70	1.86	9.4
S1 (10^5^ kN/m)	2.00	3.29	64.5
S5 (10^5^ kN/m)	2.00	3.43	71.5

**Table 4:** The modal frequencies and MAC values from the experiment, the initial FE model, and the updated FE model.

Mode	Mode Type	Experimental (Free–Ambient)	Initial FE Model			Updated FE Model		
		*f_ef_* (Hz)	*f_a,ini_* (Hz)	Difference (%)	MAC	*f_a,up_* (Hz)	Difference (%)	MAC
1	1st↔	0.648	0.601	7.25	0.973	0.634	2.18	0.978
2	2nd↔	1.219	1.182	3.04	0.977	1.196	1.92	0.983
3	3rd↔	2.186	2.201	−0.69	0.926	2.191	−0.24	0.962
4	1st↕	3.267	3.264	0.09	0.985	3.234	1.00	0.996
5	4th↔	3.493	3.595	−2.92	0.964	3.558	−1.85	0.986
6	2nd↕	3.783	3.788	−0.13	0.986	3.757	0.70	0.990
8	4th↕	4.452	4.421	0.70	0.969	4.461	−0.20	0.973
9	5th↔	4.917	5.211	−5.98	0.958	5.127	−4.28	0.989
10	5th↕	5.045	5.015	0.59	0.969	5.047	−0.04	0.986
11	1st↺	5.309	5.717	−7.69	0.865	5.372	−1.18	0.929
12	6th↕	6.027	5.966	1.01	0.911	5.966	1.02	0.889
13	6th↔	7.455	7.554	−1.33	0.499	7.497	−0.56	0.585
14	2nd↺	8.420	9.290	−10.33	0.701	9.043	−7.40	0.791
15	7th↔	9.329	10.180	−9.12	0.649	10.121	−8.49	0.828
